# Understanding variability in full-term newborns’ fNIRS data: the impact of birth weight and gestational age on infants’ speech perception abilities

**DOI:** 10.1117/1.NPh.13.3.035001

**Published:** 2026-07-06

**Authors:** Noémi Petra Szeberényi, Judit Gervain, Jessica Gemignani

**Affiliations:** a University of Padua, Department of Developmental Psychology and Socialisation, Padua, Italy; b Budapest University of Technology and Economics, Department of Cognitive Science, Budapest, Hungary; c Padova Neuroscience Center, Padua, Italy; d Université Paris Cité & CNRS, Integrative Neuroscience and Cognition Center, Paris, France

**Keywords:** newborns, perinatal physiological measures, prenatal language experience, hemodynamic response, near-infrared spectroscopy

## Abstract

**Significance:**

Language acquisition is a complex process already influenced by prenatal neural development and auditory experiences. From the onset of the third trimester, fetuses perceive sounds already influencing the fetal brain.

**Aim:**

The study investigates how the length of intrauterine language exposure, indexed by gestational age (GA), and overall maturation, indexed by birth weight (BW), affect full-term newborns’ brain responses to linguistic stimuli.

**Approach:**

Data from 14 near-infrared spectroscopy studies testing responses to different auditory sound patterns in 192 0- to 7-day-old newborns were pooled together and analyzed to assess the impact of GA and BW on changes in oxygenated hemoglobin (HbO) and deoxygenated hemoglobin (HbR).

**Results:**

Results showed that, for HbO, activations in both considered conditions were larger in the temporal than in the frontal areas, irrespective of BW or GA. A similar spatial pattern was observed for HbR, with stronger responses in the temporal compared with frontal regions across conditions. In contrast, when considering effect sizes and reflecting discrimination abilities, these were more strongly associated with BW in the bilateral frontal regions, whereas in the bilateral temporal regions, they were more strongly associated with GA.

**Conclusions:**

The findings suggest a differential impact of BW and GA on neural measures of linguistic sensitivity in newborns, reflecting their roles in biological maturation and auditory experience, respectively. Overall, the study suggests that both the length of prenatal experience and maturation play significant roles in shaping newborns’ hemodynamic responses.

## 1 Introduction

### 1.1 Measuring Neural Responses in Newborns: Near-Infrared Spectroscopy

Functional near-infrared spectroscopy (fNIRS) is a particularly useful technique to measure newborns’ brain responses, specifically the changes in the concentrations of oxygenated and deoxygenated hemoglobin associated with localized neural activation. Compared with adults, newborns and infants have thinner skin and skull and minimal hair, which promotes better contact between the head and the sensors and allows near-infrared light to penetrate deeper into the cortex.^1^^–^^5^ These features have made fNIRS a method of choice for investigating early cognitive and perceptual abilities, even within the first days of life.

At the same time, interpreting infant fNIRS data remains challenging because the developmental trajectory of the hemodynamic response function (HRF) is not yet fully characterized,^6^ particularly in newborns, where brain function and neurovascular coupling are still undergoing rapid maturation.

A key open question concerns how early hemodynamic responses are shaped by individual differences in biological maturation and prenatal experience. In newborn infants, these differences are particularly important, as they are directly associated with overall brain development, functional maturation, and later developmental outcomes. Importantly, even within typical, full-term development, substantial variability exists along both dimensions. Understanding how gestational age (GA) and birth weight (BW) influence the HRF is essential for interpreting fNIRS data and drawing informed conclusions about early neural functioning and development. The present study addresses this issue by examining their influence on fNIRS responses elicited by a range of auditory and speech perception tasks in 0- to 7-day-old newborn infants.

### 1.2 Effects of Maturation and Learning

When investigating how perinatal physiological factors influence newborns’ brain responses, speech perception provides a particularly informative model. The auditory system becomes functional between the 18th and 24th week of gestation, when the cochlea and the peripheral structures of the auditory system establish neural connections with the relevant subcortical and cortical areas.^7^^,^^8^ From this point onward, fetuses are exposed to a rich intrauterine soundscape, including the mother’s heartbeat, blood flow, and breathing; digestive and movement-related sounds; maternal speech; and salient external noises.^9^^–^^11^

These sounds are substantially filtered by the maternal tissues and the amniotic fluid, resulting in acoustic characteristics that differ from those of the broadband postnatal input. In particular, the mother’s voice reaches the fetus in different ways, as it is transmitted through air when coming from the outside, while through the inside, it is transmitted more directly through tissues and fluids, as well as in the form of vibrations, so it reaches the fetus more effectively than many other sounds.^10^ So, speech and language are some of the most prominent auditory inputs a fetus encounters during the intrauterine period.

GA determines the duration of this prenatal exposure, making it a useful proxy for assessing experience-dependent influences on the HRF and on fNIRS measures of early linguistic processing. BW, in turn, is a widely used indicator of overall prenatal health and maturation and is a strong predictor of later developmental outcomes.^12^^–^^14^ In general, both GA and BW are significant predictors of later development because they reflect the pace and completeness of early neurodevelopment. Although their effects are most evident for extremely preterm birth^12^ or very low birth weight,^15^^–^^17^ they likely shape neural development for all infants. The final weeks of gestation are characterized by rapid brain growth and organization, including substantial increases in cortical gray and white matter volumes that support emerging cognitive and language functions. Accordingly, variations in GA and BW, even within the full-term range, index meaningful differences in brain maturation. Additional time for growth *in utero* supports more mature neurovascular coupling and cerebral oxygenation, as the lung enters its final maturational stage around 36 weeks of gestation,^18^ with potential consequences for the HRF and fNIRS measures observed in full-term newborns.^18^^,^^19^ Infants with longer gestational age and higher birth weight, even within the normative range of 37 to 42 weeks, benefit from extended prenatal maturation and longer exposure to linguistic stimuli.

As a consequence, GA and BW, both readily available measures when testing newborns, can underlie substantial physiological variability even within full-term, typically developing samples. Treating such samples as homogeneous may therefore contribute to unexplained variability in developmental neuroimaging studies, particularly those relying on hemodynamic measures.

These perinatal factors not only influence overall maturation and experience but also likely shape the localization of the neural circuits underlying early language processing. From a neurobiological perspective, although the adult brain’s language system, primarily involving the superior temporal, inferior frontal, and parietal lobes in the left hemisphere, is well-characterized,^20^^,^^21^ its developmental trajectory in the infant brain is much less well understood.

Existing fNIRS studies have shown that the brain’s specialization for language begins before birth and that it is shaped significantly by prenatal auditory experience. Already at birth, infants exhibit stronger and/or more lateralized neural activity in the left temporal and frontal regions in response to native language stimuli^22^^–^^26^ than to unfamiliar ones, which in turn elicit weaker and more bilaterally distributed responses.^23^ These findings suggest that prenatal auditory exposure contributes to the specialization of language circuits and may induce long-term changes in neural dynamics. Complementary evidence from electroencephalography (EEG) studies supports this view: newborns exposed to their native language show elevated neural activity, including enhanced long-range temporal correlations in the theta band (4 to 8 Hz),^27^^,^^28^ whereas rhythmically unfamiliar languages do not produce this effect. Together, these results indicate that prenatal auditory experience shapes the neural architecture supporting language processing.^27^^,^^28^

In summary, early language exposure shapes newborns’ cognitive development and neural activation patterns. It influences auditory cortex maturation, establishes early memory traces, and fine-tunes neural responses. Investigating how perinatal factors, such as gestational age and birth weight, modulate these processes, and particularly how they modulate fNIRS responses acquired during language tasks, provides valuable insights into the organization and developmental trajectories of the infant brain.

### 1.3 Current Study

The current study aims to investigate the effects of perinatal factors on newborns’ fNIRS responses to speech-like and other sound stimuli with a meta-analytic approach pooling together data from 14 published and unpublished newborn fNIRS studies. In these studies, infants are presented with two or more conditions of different auditory stimuli, testing newborns’ abilities to discriminate among the different sounds.

In our study, we hypothesize a positive correlation between newborns’ GA/BW and their hemodynamic responses. Specifically, we correlated two different fNIRS measures with the perinatal variables: (i) the absolute magnitude of the fNIRS response in a given experimental condition, compared with baseline, and (ii) the differential fNIRS response, i.e., the difference between the responses to two conditions. We assume that the absolute magnitude of an infant’s fNIRS response is more closely related to the maturity of the infant’s overall hemodynamic activity and neurovascular coupling, whereas the differential response between two conditions represents the infant’s discrimination ability and perceptual sensitivity and may thus be more specifically linguistic. Under this hypothesis, if gestational age and/or birth weight correlate with the overall magnitude of the fNIRS response, it will be interpreted as a maturational effect, whereas if correlations are found between gestational age and/or birth weight and discrimination abilities, then more likely that they are not driven by maturation alone but also by the length of prenatal experience. We acknowledge, however, that maturation and prenatal experience are related to one another and cannot completely be unconfounded in the studies included in our analysis, as they did not explicitly manipulate these factors. We use this approach as a suggestive interpretative framework.

## 2 Materials and Methods

### 2.1 Data

#### 2.1.1 Studies

Data were assembled from 14 published and unpublished fNIRS studies conducted by our research group in two different laboratories in two different countries. The criteria for inclusion were (1) the examination of linguistic abilities of typically developing newborns, (2) not older than 7 days, (3) with the usage of the NIRS brain imaging technique, and (4) the availability of the infants’ gestational age and birth weight. In the case of papers that included more than one study, the individual experiments were considered as separate studies. This way, in the current research, 14 studies were considered from the published papers of Marino et al.,^29^ Abboub et al.,^30^ Bouchon et al.,^31^ Martinez-Alvarez et al.,^32^ and Benavides and Gervain,^33^ and from the unpublished PhD dissertation of Bouchon.^34^

Overall, the included studies comprised data from 192 newborns, aged 0 to 7 days (mean: 2.05, standard deviation: 1.1), tested in two different countries, Italy and France. Table 1 provides information about the individual sample sizes and descriptive information of each study. For further details, we refer the reader to each study’s original publication.

All studies used similar methodology testing solely auditory stimulation, with similar NIRS devices, optode configurations, and experimental designs, which made them comparable.

**Table 1:** List of studies included in the current research.

ID	Study	Publication	Condition 1	Condition 2	Language	Modality of input	Type of input	Sample size	Lab
1	1-0m-FrenchProsody-ItaBabies	Marino et al. (2025)	French standard	French deviant	Italian	Auditory	Linguistic	25	Padua
2	2-AAB-ABC-0m-Speech-ReplicationNIRx	Bouchon (2014) (PhD thesis)	ABC	AAB	French	Auditory	Linguistic	24	Paris
3	3-ABB-ABC-0m-Speech-CV	Bouchon et al. (2015)	ABC	ABB	French	Auditory	Linguistic	24	Paris
4	4-ProsodicGrouping-0m-Exp1_Duration-R=I	Abboub et al. (2016)	No contrast	Iambic	French	Auditory	Linguistic	18	Paris
5	5-ProsodicGrouping-0m-Exp1_Duration-R=T	Abboub et al. (2016)	No contrast	Trochaic	French	Auditory	Linguistic	18	Paris
6	6-ProsodicGrouping-0m-Exp2_Intensity-R=I	Abboub et al. (2016)	No contrast	Iambic	French	Auditory	Linguistic	18	Paris
7	7-ProsodicGrouping-0m-Exp2_Intensity-R=T	Abboub et al. (2016)	No contrast	Trochaic	French	Auditory	Linguistic	18	Paris
8	8-ProsodicGrouping-0m-Exp3_PitchMonolingual-R=I	Abboub et al. (2016)	No contrast	Iambic	French	Auditory	Linguistic	18	Paris
9	9-ProsodicGrouping-0m-Exp3_PitchMonolingual-R=T	Abboub et al. (2016)	No contrast	Trochaic	French	Auditory	Linguistic	18	Paris
10	10-ProsodicGrouping-0m-Exp4_PitchBilingual-R=I	Abboub et al. (2016)	No contrast	Iambic	French	Auditory	Linguistic	18	Paris
11	11-ProsodicGrouping-0m-Exp4_PitchBilingual-R=T	Abboub et al. (2016)	No contrast	Trochaic	French	Auditory	Linguistic	18	Paris
12	12-ProsodicViolation-0m-Martinezetal2023	Martinez et al. (2023)	Standard	Deviant	French	Auditory	Linguistic	25	Paris
13	13-ProsodicViolation-0m-Benavides&Gervain2017-Exp1	Benavides and Gervain (2017)	Standard	Deviant	French	Auditory	Linguistic	20	Paris
14	14-ProsodicViolation-0m-Benavides&Gervain2017-Exp2	Benavides and Gervain (2017)	Standard	Deviant	French	Auditory	Linguistic	20	Paris

#### 2.1.2 Materials

The studies examined different auditory and speech perception abilities including rule learning, prosodic grouping, and the detection of prosodic violations. Accordingly, the studies used different sets of auditory stimuli. The studies that tested rule learning compared repetition-based (e.g., ABB: “mubaba” and “penana”) and diversity-based (ABC: “mubage” and “penaku”) regularities.^31^^,^^34^ The prosodic grouping study tested sequences of pure tone stimuli that were either consistent or inconsistent with the pitch, durational, or intensity contrasts found in the prosody of the newborns’ native language.^30^ Studies assessing the detection of prosodic violations used word sequences with well-formed or ill-formed prosodic contours.^29^^,^^32^^,^^33^

For each study, fNIRS responses were investigated according to three comparisons of interest: (i) responses in condition 1 versus the zero baseline, corresponding to the less complex, less marked condition (e.g., non-repetition, no contrast, or control condition), and (ii) responses in condition 2 versus the zero baseline, corresponding to the experimental or repetition condition. For these two comparisons, absolute activations were computed to capture the overall magnitude of the hemodynamic response, which may primarily reflect general maturational effects. Finally, (iii) responses in condition 2 versus 1, quantifying the relative difference between experimental and control conditions. For this comparison, effect sizes were used to more directly index infants’ discrimination or linguistic sensitivity. Using both metrics allows us to capture complementary aspects of the fNIRS responses: absolute activations reflect the overall magnitude of the hemodynamic response and indicate how strongly the brain responds to each condition individually, whereas effect sizes compare responses among conditions, and so they index the sensitivity to the contrasts targeted by each experiment.

#### 2.1.3 Procedure

**Fig. 1:**
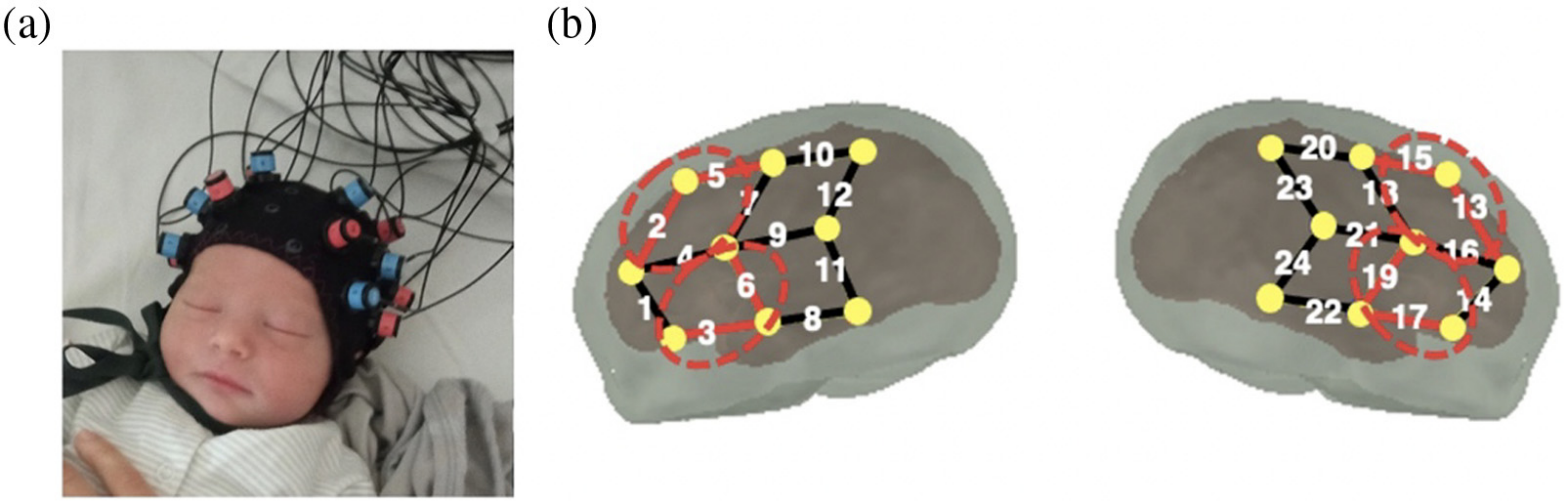
(a) NIRS headgear on a newborn. (b) Optode arrangement employed in the studies included 8 or 10 sources and 8 detectors, forming a total of 20 or 24 channels. The region of interest that this study focused on are the left temporal lobe, comprising channels 3 and 6; the left frontal lobe, comprising channels 2 and 5; the right temporal lobe, formed by channels 17 and 19; and the right frontal lobe, formed by channels 13 and 15. The relevant ROIs are highlighted in red.

In all studies, newborns were tested using a NIRx NIRS device [Fig. 1(a)], while auditory linguistic stimuli were presented via loudspeakers. Studies 2 to 14 used a NIRScout 816 machine, whereas study 1 used a NIRSport2 (source–detector separation: 3 cm; two wavelengths of 760 and 850 nm, sampling frequencies ranging between 10 and 20.32 Hz).

Eight to 10 sources and 8 detectors were positioned on newborns’ head bilaterally, with a source–detector distance of 2.5 to 3 cm, creating 10 to 12 channels on each hemisphere. Channel localization and numbering were consistent across the studies [Fig. 1(b)]. The exact anatomical localization of the resulting array is detailed in Abboub et al.^30^

As temporal and frontal regions of the cortex are known to be central in auditory and speech processing already at birth,^22^^,^^33^^,^^35^^–^^38^ the analyses focused on the left temporal lobe, defined as the cluster of channels 3 and 6, and the left inferior frontal lobe formed by channels 2 and 5 as well as the analogous right hemispheric regions [right temporal lobe: channels 17 and 19, right inferior frontal lobe: channels 13 and 15; Fig. 1(b)]. Because all studies used the same headgear configuration, channel layouts were comparable across studies, facilitating the use of these predetermined regions of interest.

### 2.2 Data Analysis

#### 2.2.1 fNIRS preprocessing

fNIRS data were pre-processed in the same way as in the original publications, which was similar across most studies. Briefly, light intensities were converted to optical densities then to hemoglobin concentration changes using the modified Beer–Lambert law with the absorption coefficients μa, ${\mathrm{mm}}^{-1}\times {\mathrm{mM}}^{-1}$: μa (HbO,760  nm)=0.1496, μa(HbO,850  nm)=0.2526, μa(HbR,760  nm)=0.3865, and μa(HbR,850  nm)=0.1798. The product of the optical pathlength and the differential pathlength factor was set to 1, resulting in concentration changes being expressed in mM×mm. A bandpass filter between 0.01 and 0.7 or 1 Hz—depending on the considered research paper—was applied to concentration changes using an *fft* digital filter. Then, as illustrated in Gemignani and Gervain,^39^ blocks of single-trial data were rejected if they contained motion artifacts or if the light intensity reached the saturation value, with motion artifacts defined as signal changes larger than 0.1  mM×mm over 0.2 s. The artifact detection and trial rejection procedure were performed independently for each channel, and channels with less than at least two valid blocks were discarded from the analysis. Trial inclusion rate for each study ranged between 50 and 72% (across studies, mean: 60% and std: 8%). Finally, for the non-rejected blocks, a baseline was linearly fit between the mean of the 5 s preceding the onset of the block and the mean of the 5 s preceding the onset of the next one. This pre-processing routine has been shown to yield an accurate recovery of the infant hemodynamic response.^39^^,^^40^

#### 2.2.2 Activation values and effect sizes

Individual trial data were employed to compute infant-level activation values, i.e., response magnitude, for the comparison of each condition against the zero baseline (condition 1 versus 0 and condition 2 versus 0) as well as infant-level effect sizes, i.e., discrimination ability, for the between-condition comparisons (condition 1 versus 2). Activation values for each condition were computed as the average of each non-rejected trial response within a time window starting at the onset of stimulation and lasting up to 15 s after the stimulus’ end; thus, the window length differed among studies as they employed different windows. For each newborn, channel, and hemoglobin component, obtained activation values were then averaged across trials if at least two valid, non-rejected trials were available; otherwise, that channel was rejected. For each newborn, activation values were then further averaged within the regions of interest (ROIs) described in Sec. 2.1.3.

**Fig. 2:**
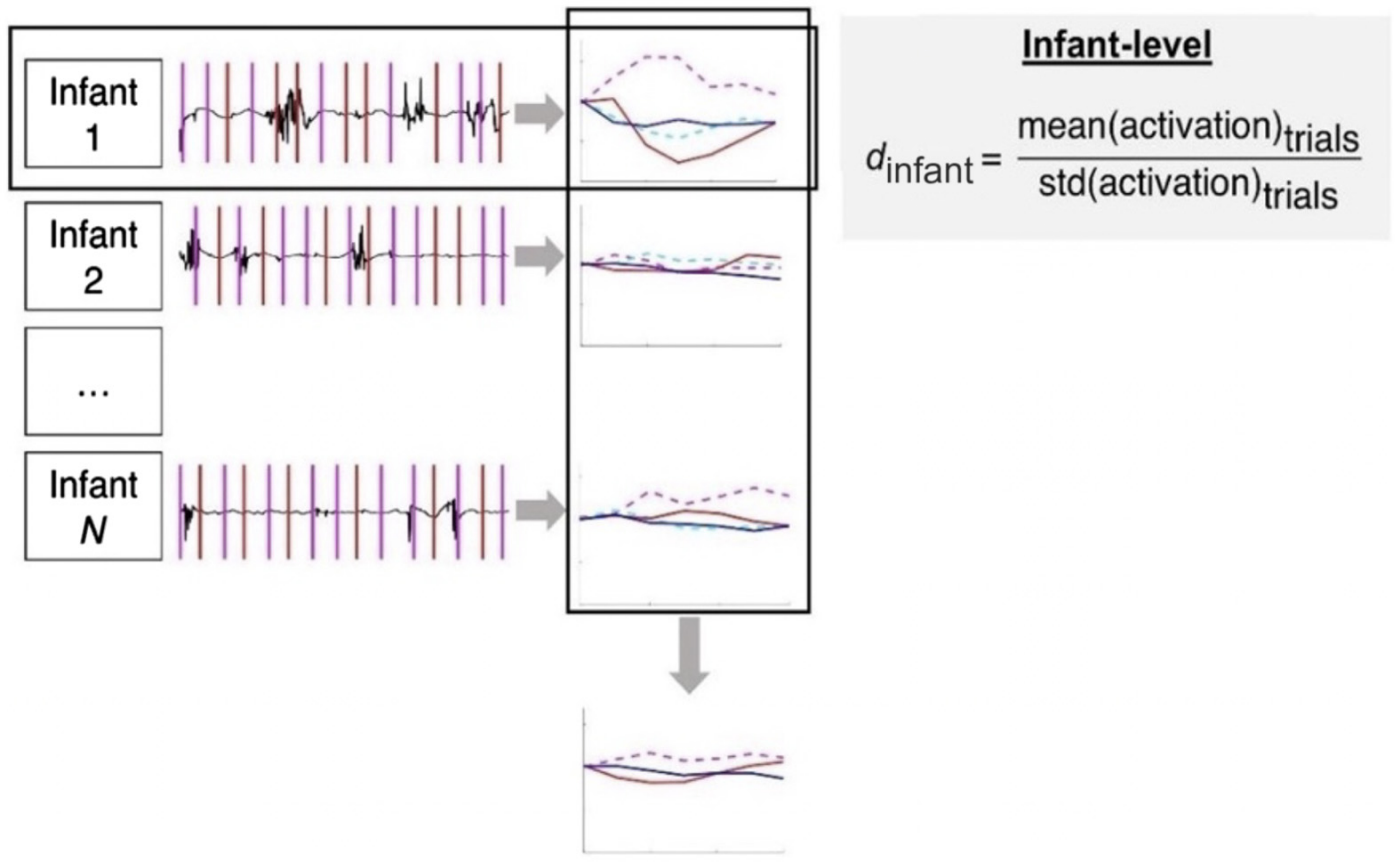
Schematic representation of infant-level effect size calculation. Activation refers to the condition 1 versus 0, condition 2 versus 0, and condition 2 versus 1 contrasts computed as the average of the hemodynamic response function along its time course. Magenta and cyan indicate repetition trials (HbO and HbR, respectively), and red and blue indicate non-repetition trials (HbO and HbR, respectively).^6^

Infant-level effect sizes of between-condition differential responses, i.e., discrimination, were obtained by calculating the difference between the average activations in conditions 2 and 1 and dividing the result by the standard deviation of trial-wise activation values from both conditions (Fig. 2). Then, for each baby and hemoglobin component, channel-wise effect sizes were averaged within the four ROIs.

These ROI-based analyses were used to examine the effects of maturation and experience within language-related circuitry. To assess whether these effects were specific to these regions or more broadly distributed, we conducted additional single-channel analyses across the full array as well as those channels that are included in the ROIs to evaluate the consistency of the results with the ROI-averaged approach described above.

#### 2.2.3 Extraction of individual gestational age and birth weight data

For each newborn, information about gender at birth, date of birth, date of test, gestational age, and birth weight was collected. For the purposes of all subsequent analyses, birth weight was registered in grams, and gestational age in days. Some of this information was missing for 90 newborns, who thus needed to be excluded from the overall sample of 282 newborns, resulting in a final dataset of 192 participants.

Gestational ages ranged from 255 to 298 days (M=277.9, SD=8.44) and birth weight from 2235 to 4535 g (M=3308, SD=387.25). As expected, there was a statistically significant correlation between the two (rho=0.3968, p<0.001).

#### 2.2.4 Statistical analysis

##### 2.2.4.1 Linear Mixed-Effects Modeling

Linear mixed-effects models with fixed factors age in days, gestational age at birth, birth weight, ROI (temporal/frontal), and hemisphere (LH/RH) were run separately over the three dependent variables of interest (condition 1 activation, condition 2 activation, and effect size of the condition 2 versus 1 differential response) using oxygenated hemoglobin (HbO) and deoxygenated hemoglobin (HbR). The random effects structure included random intercepts for subject, subject:ROI, and study. We first selected the random effects structure. In case of failure to converge, the structure was gradually simplified by removing the random intercept for study first, followed by subject:ROI, and then subject. We then performed model selection. Candidate fixed effects were added one at a time to the model, and the resulting models were compared. For each analysis, the model with the lowest AIC value was selected as the best-fitting model, or, when multiple models showed comparable AIC values, we selected the most parsimonious model. Single-channel analyses followed the same procedure. Their model specifications and resulting models are reported in Tables S1 and S2 in the Supplementary Material.

## 3 Results

### 3.1 Analysis of ROI-Averaged Activations and Effect Sizes

#### 3.1.1 Oxygenated hemoglobin

**Fig. 3:**
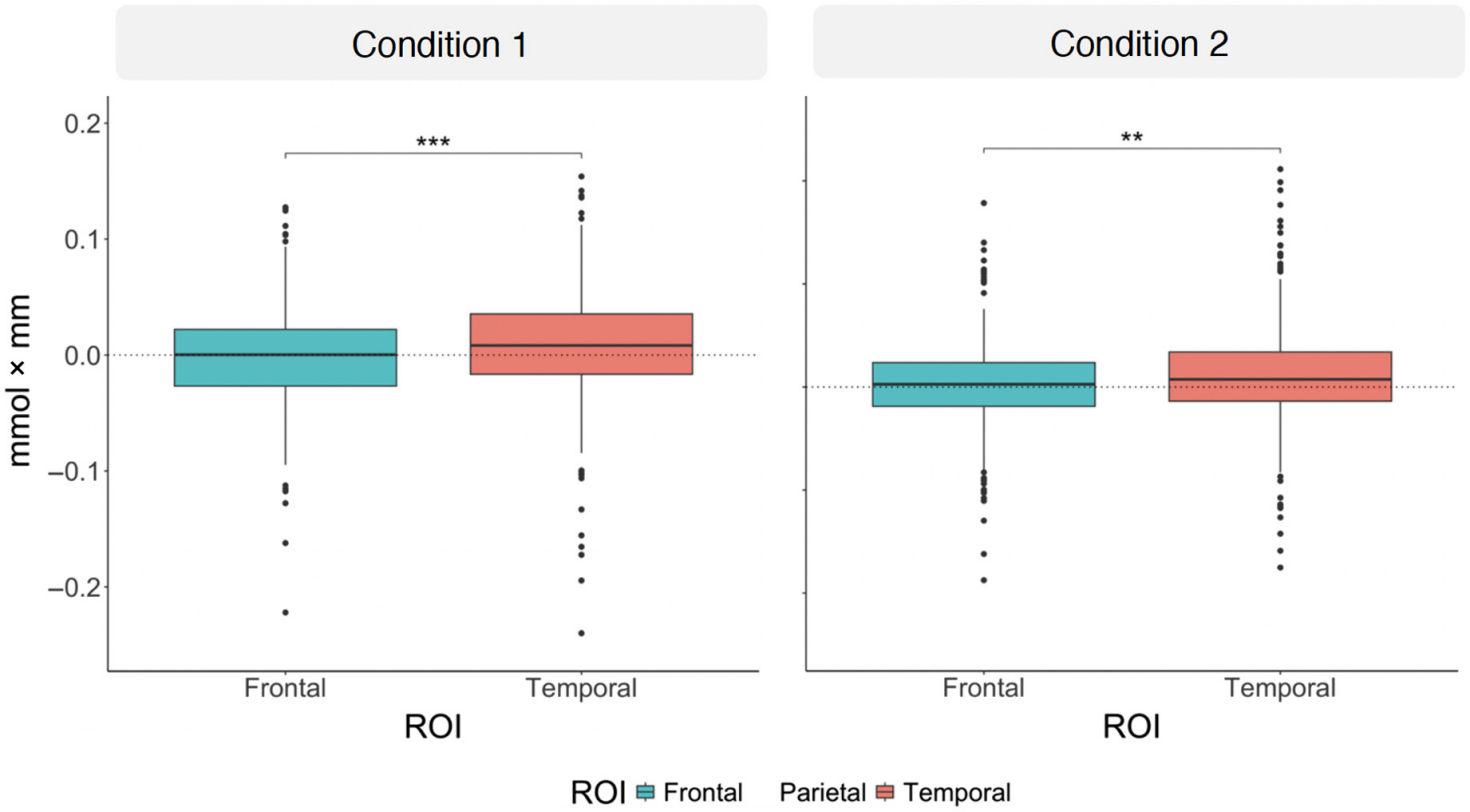
Distributions of HbO activations elicited by conditions 1 and 2 in the two ROIs, the temporal and frontal: for both, the analysis showed the main effect of ROI, driven by larger activations in the temporal area compared with the frontal area.

For condition 1 activations against baseline, the best-fitting model included a random intercept for subject and fixed effect for ROI. It yielded a significant main effect of ROI (F(1,532)=10.96, p<0.001). The *post hoc* comparison showed this was due to larger activations in the temporal than in the frontal area (mean difference=0.00957, SE=0.00289, t(527)=3.3, p<0.001, Fig. 3).

For condition 2 activations against baseline, the best-fitting model included a random intercept for subject and subject:ROI and a fixed effect of ROI. This model yielded a significant main effect of ROI (F(1,177)=8.43, p<0.01), carried by greater activations in the temporal than in the frontal area (mean difference=0.00902, SE=0.00311, t(171)=2.9, p<0.01, Fig. 3).

For the effect sizes of the differential activation between conditions 1 and 2, the best-fitting model included a random intercept for subject and fixed effect of gestational age but yielded no significant effects.

#### 3.1.2 Deoxygenated hemoglobin

**Fig. 4:**
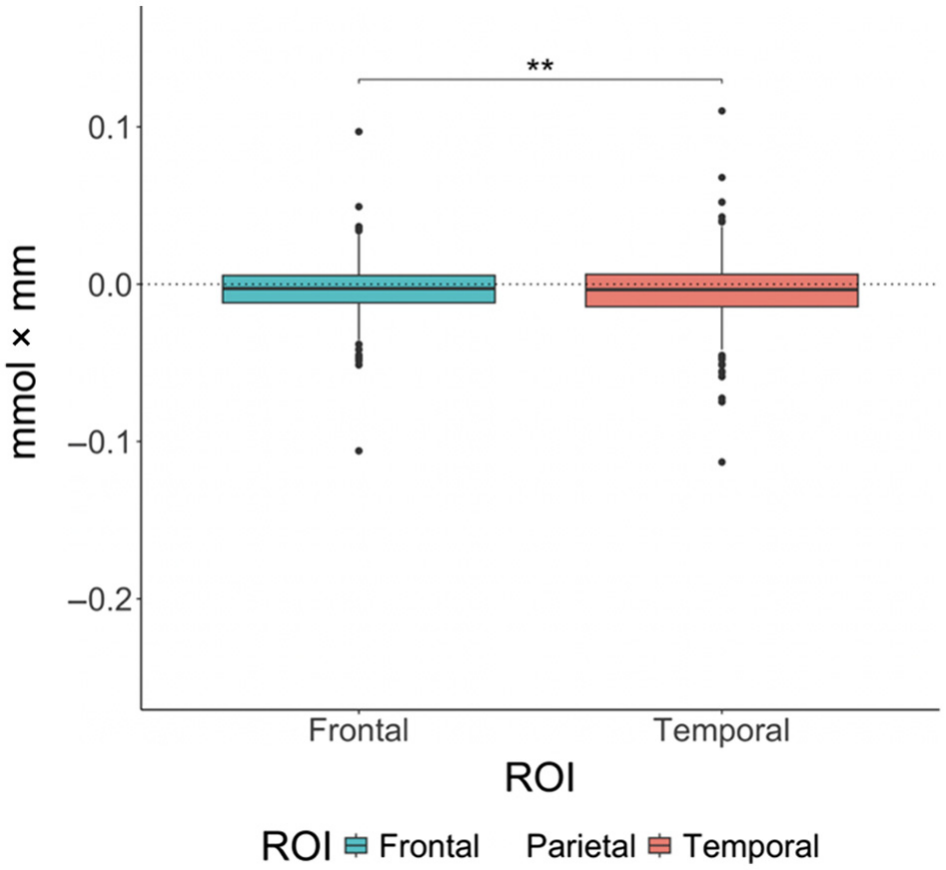
Distributions of HbR activations elicited by condition 1 in the two ROIs, the temporal and frontal: the analysis showed the main effect of ROI, driven by larger negative activations in the temporal area compared with the frontal area.

For condition 1 activations against baseline, the best-fitting model included a random intercept for subject and fixed effect for ROI. This model yielded a significant main effect of ROI (F(1,537)=8.22, p<0.01), carried by greater, i.e., more negative, activations in the temporal than in the frontal area (mean difference=0.00352, SE=0.00123, t(536)=2.866, p<0.01, Fig. 4).

For condition 2 activations, the best-fitting model included random intercepts for subject and subject:ROI and fixed effects of ROI but yielded no significant effects or interactions.

**Fig. 5:**
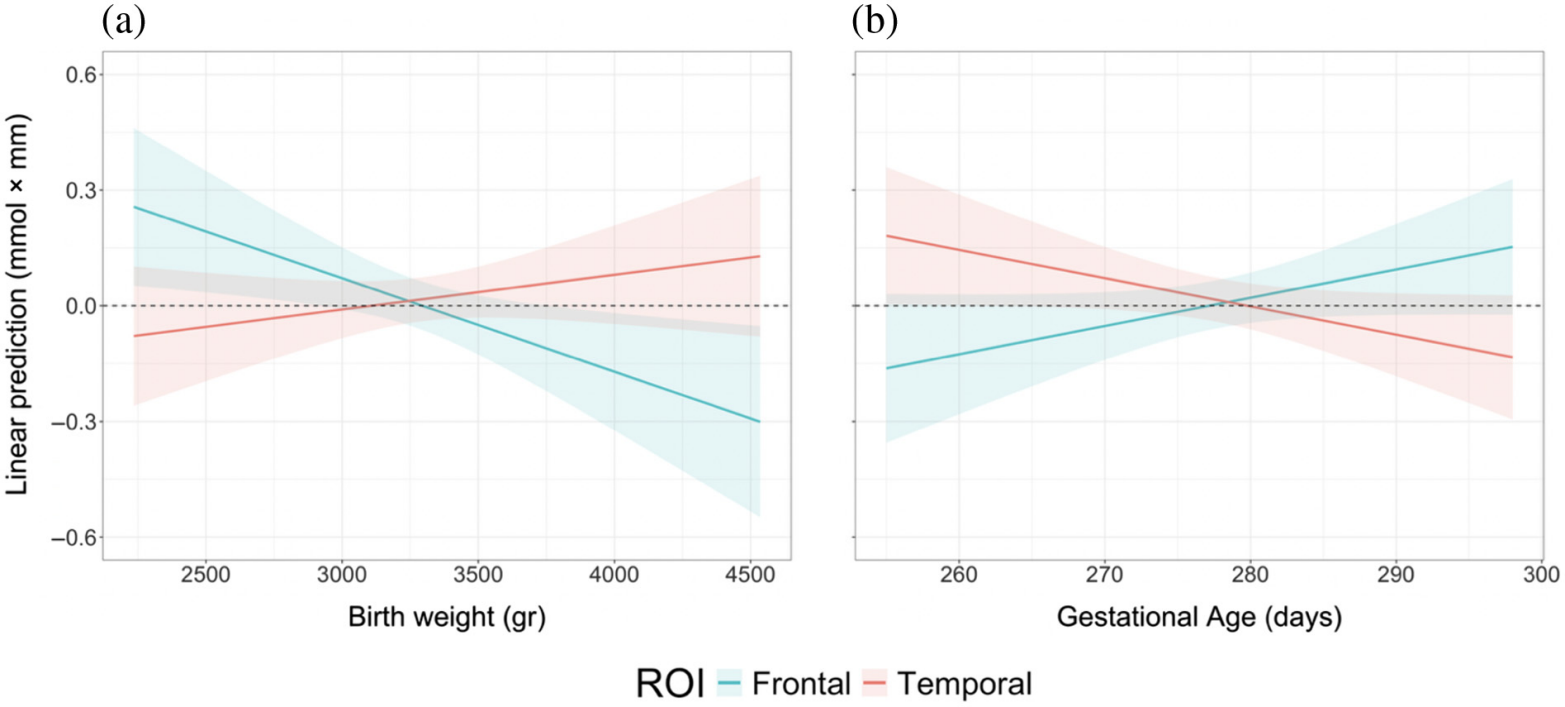
(a) Interaction of BW and ROI showing a significantly more positive slope in the temporal than in the frontal areas (p<0.01) for the effect sizes of the differential response between conditions 1 and 2 in HbR. (b) Interaction of GA and ROI yielding a significantly more positive slope for the frontal than for the temporal areas (p<0.01) for the effect sizes of the differential response between conditions 1 and 2 in HbR.

For the effect sizes of the differential response between conditions 1 and 2, the best-fitting model included a random intercept for subject and subject:ROI and fixed effects for birth weight, ROI, and gestational age. The model yielded a significant main effect of ROI (F(1,160)=7.03, p<0.01), a significant BW × ROI interaction (F(1,177)=10.39, p<0.01), and a significant ROI × GA interaction (F(1,162)=10.87, p<0.01). The significant main effect of ROI was due to more positive effect sizes, i.e., less strong effects in the temporal than in the frontal areas, which, however, did not hold significance following *post hoc* analyses. For the BW × ROI interaction, the *post hoc* analysis revealed that the estimated slopes were found to be significantly more negative in the frontal than in the temporal ROIs with increasing birth weight (mean difference frontal−temporal=−0.000332, SE=0.000103, t(176)=−3.22, p<0.01) [Fig. 5(a)]. For GA × ROI, slopes were significantly more negative in the temporal than in the frontal ROIs with increasing gestational age (mean difference frontal−temporal=0.0147, SE=0.00445, t(162)=3.29, p<0.01) [Fig. 5(b)].

### 3.2 Single-Channel Analyses

The analysis of single-channel data for the whole array of channels yielded the main effect of ROI, with larger involvement of the temporal area than other areas for condition 1 versus 0 as well as condition 2 versus 0 over HbR (Table S1 in the Supplementary Material for the statistics; Figs. S1 and S2 in the Supplementary Material). Furthermore, in condition 1 versus 0, the RH showed stronger responses than the LH (Table S1 in the Supplementary Material for the statistics; Figs. S1 and S2 in the Supplementary Material).

The analysis of single-channel data from the channels included in the ROIs under investigation, the main effect of ROI was found in condition 1 versus 0 as well as in condition 2 versus 0 over HbO and HbR (Table S2, Figs. S3 and S4 in the Supplementary Material). In addition, the interaction among BW, GA, and ROI (Fig. 5) was significant over HbR effect sizes for the condition 1 versus 2 comparison (Fig. S5 in the Supplementary Material).

## 4 Discussion

### 4.1 Effects of Birth Weight and Gestational Age on Brain Responses to Language

In the case of oxygenated hemoglobin activations to both condition 1, i.e., the control condition, and condition 2, i.e., the experimental condition, the analysis found no significant correlations with either BW or GA but rather larger overall activations in the bilateral temporal areas compared with the frontal ones, a finding with ample convergence with the literature on newborns’ early perceptual and linguistic abilities.^35^^,^^41^^,^^42^

Interestingly, measures derived from HbR presented a more diversified pattern. Although activations in either condition, taken separately, did not show an association with either BW or GA, they do display larger, as in, more negative, activations in the temporal areas compared with the frontal ones only for condition 1. When interpreting these results, a factor to consider relates to the nature of the dataset. Although condition 2 represents the experimentally manipulated contrast, it does not reflect a homogeneous underlying cognitive process across studies, as different datasets targeted distinct linguistic abilities. In contrast, condition 1 indexes a more naturalistic response to speech, which is robustly elicited across paradigms. As a consequence, when considering activations in the two conditions separately from one another, individual differences may be more consistently expressed in condition 1, whereas effects in condition 2 may be diluted by variability in task demands and targeted linguistic processes.

However, when moving beyond activations and investigating between-conditions effect sizes, which reflect infants’ discrimination abilities and thus are more inherently correlated to the linguistic mechanisms under investigation, their analysis revealed statistically significant interactions between ROI and BW, and ROI and GA, with increasing BW associated with stronger activations in bilateral frontal areas, and increasing GA associated with stronger activations in bilateral temporal areas.

Following our interpretative proposal that GA indicates the length of the prenatal experience, and BW represents the extent of biological maturation, these findings suggest that GA exhibits a stronger effect on a perceptual, auditory level affecting auditory discrimination mainly; hence, it is significantly associated to stronger activations of the temporal areas where the auditory cortex is located. On the other hand, BW is suggested to reflect the maturation and development of more abstract, linguistic brain structures, i.e., Broca’s area, located in the frontal region. This interpretation is further supported by evidence from developmental neuroimaging studies showing distinct maturation timelines for temporal and frontal cortical regions. Functional studies in preterm and term infants consistently report robust activation of temporal auditory regions in response to speech and phonemic contrasts early in development, even at very low gestational ages.^43^^,^^44^ In contrast, frontal regions are also recruited during early speech processing but show more selective, condition-dependent activation patterns, rather than broad responsiveness.^43^ Structural and longitudinal MRI studies further indicate that frontal cortices follow a more protracted maturational trajectory compared with primary and secondary temporal regions, with continued postnatal development of cortical organization and connectivity.^44^^,^^45^ Importantly, gestational age has been shown to positively correlate with prosody-based speech discrimination responses specifically in anterior temporal regions at term-equivalent age, suggesting that longer prenatal exposure supports the maturation of prosodic processing.^46^ According to this framework, we hypothesize that gestational age, indexing the duration of prenatal experience, preferentially modulates temporal cortex responses, whereas birth weight, indexing overall biological maturation, is more strongly associated with frontal cortical activation, providing a developmental account for the opposing BW and GA patterns observed in our data.

### 4.2 Implications

In the present dataset, analyses of activations in the two conditions considered separately did not reveal consistent effects of birth weight or gestational age. In contrast, clear effects emerged when considering the differential response conditions, i.e., the effect size indexing sensitivity to the linguistic manipulation. This pattern suggests that developmental variability is more closely reflected in measures indexing task-related performance, with effect sizes capturing infants’ sensitivity to the linguistic contrast and thus providing an index of emerging functional ability, rather than being expressed in absolute levels of neural activation. Moreover, these effects were observed specifically within language-relevant ROIs and not in whole-array analyses, highlighting that they reflect functionally meaningful differences in neural responses within language-related circuitry rather than general activation differences.

Surprisingly, these results were found on HbR only and not HbO. This is particularly relevant as many infant studies rely mostly on HbO because it usually provides a better signal-to-noise ratio.^3^ Our findings suggest that, although HbO is more sensitive to the experimental manipulations in typical experiments with newborns, HbR may better capture broader aspects of biological maturation, growth, and prenatal experience. This pattern is likely rooted in fundamental aspects of neurovascular development in the neonatal brain. Studies in developing animals, for example, have shown that early hemodynamic responses are often dominated by increases in HbR with minimal or delayed arterial hyperemia, reflecting local metabolic demand at the capillary level before mature pial arterial blood flow emerges.^47^ Although these findings come from animal models, the principles are directly relevant to human neonates, whose vascular regulation and neurovascular coupling are still maturing. Consistent with this, human infant fNIRS studies report substantial heterogeneity in the amplitude, polarity, and temporal profile of HbO and HbR responses during sensory stimulation, indicating that vascular control mechanisms are not yet stabilized in early postnatal life.^48^ Multimodal measurements further suggest that early hemodynamic responses in infants can be dominated by local oxygen consumption and microvascular metabolism rather than by robust increases in cerebral blood flow.^49^ Complementing these findings, analyses of spontaneous low-frequency hemoglobin oscillations demonstrate systematic postnatal changes in the coordination between HbO and HbR signals (hPod), reflecting rapid maturation of vascular–metabolic coupling in the first weeks of life.^50^ Together, this evidence provides a mechanistic rationale for why HbR, by reflecting deoxyhemoglobin dynamics at the microvascular level, may be particularly sensitive to early developmental differences, including prenatal experience and postnatal maturation.

Our findings reinforce previous suggestions that HbR should be routinely included in the fNIRS analyses, especially with infant data, given its sensitivity to microvascular and metabolic maturation. The stronger correlation of HbR with BOLD signals^51^^–^^53^ further highlights its physiological relevance for interpreting functional activation in newborns.

### 4.3 Limitations

In the current research, some limitations need to be acknowledged. The first one arises from the heterogeneity of the included studies. Although all studies followed broadly consistent methodologies and experimental designs, they differed in the specific linguistic abilities they targeted and consequently in the types of auditory or speech stimuli employed. This variability may limit direct comparability across studies, but it also increases the generalizability of our findings, by highlighting patterns that are not specific to a single experimental manipulation.

A related limitation concerns the theoretical nature of conditions 1 and 2. Although condition 1 consistently involved relatively basic and naturalistic auditory or speech stimulation across studies, condition 2 varied more substantially in terms of the specific linguistic manipulation employed (e.g., phonemic, prosodic, or structural contrasts). As a consequence, the underlying cognitive processes indexed by condition 2 were less homogeneous in the dataset. This variability may have reduced sensitivity to detect systematic effects of biological maturation and prenatal experience for condition 2 at the group level. Future work based on more homogeneous experimental manipulations will be important to better isolate how maturation influences responses to specific higher-level linguistic contrasts.

One specificity of our study is that all data come from a single research group (although the different studies were conducted in different laboratories in different countries by different experimenters). This may limit generalizability to some extent. At the same time and more importantly, the methodological consistency (similar NIRS device, standardized procedures, and analysis pipelines) may also offer a key strength. Minimizing variability is essential for detecting subtle effects of maturation and experience on brain responses elicited by different language tasks. Consistent procedures across studies increase sensitivity to task-specific developmental changes in cortical responses that might otherwise be obscured in more heterogeneous datasets. Furthermore, it is increasingly acknowledged that smaller-scale or within-lab meta-analyses can provide valuable insights into moderators and sources of variability that individual studies cannot capture,^54^^–^^56^ underscoring the relevance of this approach for addressing our research question.

Finally, we note that in natural, non-medically assisted pregnancies, gestational age is an estimate, which may lack precision, potentially varying by 1 to 2 weeks. This imprecision could influence the estimate of the developmental stage of the newborns at the time of the study, thereby affecting the interpretation of its significance with respect to the linguistic abilities and maturation.

### 4.4 Future Directions

This study highlights the different roles of birth weight and gestational age in shaping newborns’ neural responses to language, suggesting several directions for future research. First, examining preterm and low birth weight newborns could further enhance the understanding of the effects of the length of prenatal experience and biological maturation. Besides, longitudinal studies could examine the developmental outcomes in regard to the examined physiological measures at birth. Finally, the generalizability of these findings may be tested by expanding the research to more diverse linguistic and cultural contexts.

Finally, an important future direction would be the characterization of the temporal dynamics between HbO and HbR responses across development. Inverted or atypical response patterns, which are common in infant fNIRS data, may reflect immature or transitioning neural response profiles, and their interpretation may benefit from analyses that consider response timing, polarity, and the covariation among hemoglobin signals. Future studies could therefore investigate how the coupling and temporal alignment of HbO and HbR responses evolve with maturation and experience and whether these features better capture developmental changes in early language processing. Combining fNIRS with complementary modalities, such as EEG, may further help disentangle how developmental changes in neural processing relate to observed hemodynamic response patterns in newborns.

## 5 Conclusion

The increasing use of fNIRS in developmental research affords the opportunity to investigate brain responses in several perceptual domains from the earliest stages of life. Yet, a critical unanswered issue is whether and how the shape of the early HRF is modulated by perinatal factors such as BW and GA. We found that increasing BW was associated with effect sizes of linguistic discrimination getting stronger in the bilateral frontal areas; these findings together support the role of BW in reflecting the extent of biological maturation. Further, increasing GA was associated with effect sizes becoming stronger in the bilateral temporal areas, suggesting that duration of prenatal experience significantly modulates auditory discrimination abilities. Relations with perinatal factors were only significant when using measures derived from the HbR component, indicating its higher sensitivity, compared with HbO, to overall biological maturation and growth.

Understanding how perinatal factors influence newborns’ HRF may provide a better understanding of the unexplained variability in developmental studies, thus contributing to achieving a better interpretation of newborns’ fNIRS data and to drawing more accurate conclusions about early neural functioning and development.

## Supplementary Material

10.1117/1.NPh.13.3.035001.s01

## Data Availability

The codes used for all statistical analyses reported in this study, along with their results, are available at https://osf.io/6mzqp. The data are available upon reasonable request to the authors.

## References

[1] Gervain J. et al., “Using functional near-infrared spectroscopy to study the early developing brain: future directions and new challenges,” Neurophotonics 10(2), 023519 (2023). 10.1117/1.NPh.10.2.023519 37020727 PMC10068680

[r2] Aslin R. N. Mehler J., “Near-infrared spectroscopy for functional studies of brain activity in human infants: promise, prospects, and challenges,” J. Biomed. Opt. 10(1), 011009 (2005). JBOPFO 1083-3668 10.1117/1.1854672 15847575

[r3] Lloyd-Fox S. Blasi A. Elwell C. E., “Illuminating the developing brain: the past, present and future of functional near infrared spectroscopy,” Neurosci. Biobehav. Rev. 34(3), 269–284 (2010). 10.1016/j.neubiorev.2009.07.008 19632270

[r4] Ayaz H. et al., “Optical imaging and spectroscopy for the study of the human brain: status report,” Neurophotonics 9(S2), S24001 (2022).2329-4248 10.1117/1.NPh.9.S2.S24001 36052058 PMC9424749

[5] Gervain J. et al., “Near-infrared spectroscopy: a report from the McDonnell infant methodology consortium,” Dev. Cogn. Neurosci. 1(1), 22–46 (2011). 10.1016/j.dcn.2010.07.004 22436417 PMC6987576

[6] Issard C. Gervain J., “Variability of the hemodynamic response in infants: influence of experimental design and stimulus complexity,” Dev. Cogn. Neurosci. 33, 182–193 (2018). 10.1016/j.dcn.2018.01.009 29397345 PMC6969282

[7] Birnholz J. C. Benacerraf B. R., “The development of human fetal hearing,” Science 222(4623), 516–518 (1983). 10.1126/science.6623091 6623091

[8] Eggermont J. J. Moore J. K., “Morphological and functional development of the auditory nervous system,” in Human Auditory Development. Springer Handbook of Auditory Research, Werner L. Fay R. Popper A., Eds., Vol. 42, pp. 61–105, Springer, New York, NY (2012).

[9] Gerhardt K. J. Abrams R. M., “Fetal exposures to sound and vibroacoustic stimulation,” J. Perinatol.: Off. J. California Perinatal Assoc. 20(8 Pt 2), 21–30 (2000). 10.1038/sj.jp.7200446 11190697

[r10] Dealessandri G. Vivalda M., “The mother’s womb acoustic environment: study of the original sounds and replication for pre-term infants,” in J. Phys.: Conf. Ser, IOP Publishing, Vol. 1075, p. 012056 (2018). 10.1088/1742-6596/1075/1/012056

[11] Parga J. J. et al., “A description of externally recorded womb sounds in human subjects during gestation,” PLoS ONE 13(5), 0197045 (2018). POLNCL 1932-6203 10.1371/journal.pone.0197045 PMC5944959 29746604

[12] Hack M. Klein N. K. Taylor H. G., “Long-term developmental outcomes of low birth weight infants,” Future Child. 5(1), 176–196 (1995). 10.2307/1602514 7543353

[r13] Boardman J. D. et al., “Low birth weight, social factors, and developmental outcomes among children in the United States,” Demography 39(2), 353–368 (2002). 10.1353/dem.2002.0015 12048956

[14] Cortese M. Moster D. Wilcox A. J., “Term birthweight and neurodevelopmental outcomes,” Epidemiology 32(4), 583–590 (2021). 10.1097/EDE.0000000000001350 34001752 PMC8439103

[15] Barre N. et al., “Language abilities in children who were very preterm and/or very low birth weight: a meta-analysis,” J. Pediatr. 158(5), 766–774 1 (2011). 10.1016/j.jpeds.2010.10.032 21146182

[r16] Wade M. et al., “Normal birth weight variation and children’s neuropsychological functioning: links between language, executive functioning, and theory of mind,” J. Int. Neuropsychol. Soc. 20(9), 909–919 (2014). 10.1017/S1355617714000745 25171131

[17] Zerbeto A. B. Cortelo F. M. Filho É.B. C., “Association between gestational age and birth weight on the language development of Brazilian children: a systematic review,” J. Pediatr. 91(4), 326–332 (2015). 10.1016/j.jped.2014.11.003 25913048

[18] Copland I. Post M., “Lung development and fetal lung growth,” Paediatr. Respir. Rev. 5, 259–264 (2004). 10.1016/S1526-0542(04)90049-8 14980282

[19] Hislop A. A. Wigglesworth J. S. Desai R., “Alveolar development in the human fetus and infant,” Early Hum. Dev. 13(1), 1–11 (1986). EHDEDN 0378-3782 10.1016/0378-3782(86)90092-7 3956418

[20] Friederici A. D., “The cortical language circuit: from auditory perception to sentence comprehension,” Trends Cogn. Sci. 16(5), 262–268 (2012). TCSCFK 1364-6613 10.1016/j.tics.2012.04.001 22516238

[21] Poeppel D., “The neuroanatomic and neurophysiological infrastructure for speech and language,” Curr. Opin. Neurobiol. 28, 142–149 (2014). COPUEN 0959-4388 10.1016/j.conb.2014.07.005 25064048 PMC4177440

[22] Peña M. et al., “Sounds and silence: an optical topography study of language recognition at birth,” Proc. Natl. Acad. Sci. U. S. A. 100(20), 11702–11705 (2003). 10.1073/pnas.1934290100 14500906 PMC208821

[r23] Sato Y. Sogabe Y. Mazuka R., “Development of hemispheric specialization for lexical pitch—accent in Japanese infants,” J. Cogn. Neurosci. 22, 2503–2513 (2010). JCONEO 0898-929X 10.1162/jocn.2009.21377 19925204

[r24] May L. et al., “Language and the newborn brain: does prenatal language experience shape the neonate neural response to speech?” Lang. Sci. 2, 222 (2011). 10.3389/fpsyg.2011.00222 PMC3177294 21960980

[r25] Vannasing P. et al., “Distinct hemispheric specializations for native and non-native languages in one-day-old newborns identified by fNIRS,” Neuropsychologia 84, 63–69 (2016). NUPSA6 0028-3932 10.1016/j.neuropsychologia.2016.01.038 26851309

[26] May L. et al., “The specificity of the neural response to speech at birth,” Dev. Sci. 21(3), 1–9 (2018). 10.1111/desc.12564 28503845

[27] Mariani B. et al., “Prenatal experience with language shapes the brain,” Sci. Adv. 9(47), eadj3524 (2023). SACDAF 2375-2548 10.1126/sciadv.adj3524 37992161 PMC10664997

[28] Barajas M. C. O. Guevara R. Gervain J., “Neural oscillations and speech processing at birth,” iScience 26(11), 108187 (2023). 10.1016/j.isci.2023.108187 37965146 PMC10641252

[29] Marino C. et al., “Do newborns detect prosodic violations in an unfamiliar language at birth?,” Brain Lang. 271, 105640 (2025). 10.1016/j.bandl.2025.105640 40946376

[30] Abboub N. Nazzi T. Gervain J., “Prosodic grouping at birth,” Brain Lang. 162, 46–59 (2016). 10.1016/j.bandl.2016.08.002 27567401

[31] Bouchon C. Nazzi T. Gervain J., “Hemispheric asymmetries in repetition enhancement and suppression effects in the newborn brain,” PLoS ONE 10(10), 1–17 (2015). POLNCL 1932-6203 10.1371/journal.pone.0140160 PMC4618998 26485434

[32] Martinez-Alvarez A. et al., “Newborns discriminate utterance-level prosodic contours,” Dev. Sci. 26(2), 13304 (2023). 10.1111/desc.13304 35841609

[33] Benavides-Varela S. Gervain J., “Learning word order at birth: a NIRS study,” Dev. Cogn. Neurosci. 25, 198–208 (2017). 10.1016/j.dcn.2017.03.003 28351534 PMC6987835

[34] Bouchon C., “Functional asymmetry between consonants and vowels from birth to 6 months of age—cerebral imaging and behavioral data,” PhD thesis, Université Paris Cité (2014).

[35] Gervain J. et al., “The neonate brain detects speech structure,” Proc. Natl. Acad. Sci. 105(37), 14222–14227 (2008). 10.1073/pnas.0806530105 18768785 PMC2544605

[r36] Gervain J. Berent I. Werker J. F., “Binding at birth: the newborn brain detects identity relations and sequential position in speech,” J. Cogn. Neurosci. 24(3), 564–574 (2012). JCONEO 0898-929X 10.1162/jocn_a_00157 22066581 PMC3270491

[r37] Telkemeyer S. et al., “Sensitivity of newborn auditory cortex to the temporal structure of sounds,” J. Neurosci.: Off. J. Soc. Neurosci. 29(47), 14726–14733 (2009). 10.1523/JNEUROSCI.1246-09.2009 PMC6666009 19940167

[38] Dehaene-Lambertz G. Dehaene S. Hertz-Pannier L., “Functional neuroimaging of speech perception in infants,” Science 298(5600), 2013–2015 (2002). SCIEAS 0036-8075 10.1126/science.1077066 12471265

[39] Gemignani J. Gervain J., “Comparing different pre-processing routines for infant fNIRS data,” Dev. Cogn. Neurosci. 48, 100943 (2021). 10.1016/j.dcn.2021.100943 33735718 PMC7985709

[40] Gemignani J. et al., “Reproducibility of infant fNIRS studies: a meta-analytic approach,” Neurophotonics 10(2), 023518 (2023). 10.1117/1.NPh.10.2.023518 36908681 PMC9997722

[41] May L. et al., “The specificity of the neural response to speech at birth,” Dev. Sci. 21(3), e12564 (2017). 10.1111/desc.12564/full 28503845

[42] Gemignani J. Gervain J., “Brain responses to repetition-based rule-learning do not exhibit sex differences: an aggregated analysis of infant fNIRS studies,” Sci. Rep. 14(1), 2611 (2024). 10.1038/s41598-024-53092-2 38297068 PMC10831066

[43] Mahmoudzadeh M. et al., “Syllabic discrimination in premature human infants prior to complete formation of cortical layers,” Proc. Natl. Acad. Sci. U. S. A. 110(12), 4846–4851 (2013). 10.1073/pnas.1212220110 23440196 PMC3607062

[44] Baldoli C. et al., “Maturation of preterm newborn brains: a fMRI–DTI study of auditory processing of linguistic stimuli and white matter development,” Brain Struct. Funct. 220(6), 3733–3751 (2015). 10.1007/s00429-014-0887-5 25244942

[45] Dubois J. Dehaene-Lambertz G., “Fetal and postnatal development of the cortex: MRI and genetics,” Brain Mapp.: Encyclopedic Ref. 2, 11–19 (2015). 10.1016/B978-0-12-397025-1.00194-9

[46] Alexopoulos J. et al., “The duration of intrauterine development influences discrimination of speech prosody in infants,” Dev. Sci. 24(5), e13110 (2021). 10.1111/desc.13110 33817911 PMC11475226

[47] Kozberg M. G. et al., “Resolving the transition from negative to positive blood oxygen level-dependent responses in the developing brain,” Proc. Natl. Acad. Sci. U. S. A. 110(11), 4380–4385 (2013). 10.1073/pnas.1212785110 23426630 PMC3600493

[48] Sato H. et al., “Cerebral hemodynamics in newborn infants exposed to speech sounds: a whole-head optical topography study,” Hum. Brain Mapp. 33(9), 2092–2103 (2012). HBMAEE 10.1002/hbm.21350 21714036 PMC6870359

[49] Nourhashemi M. et al., “Neurovascular coupling in the developing neonatal brain at rest,” Hum. Brain Mapp. 41(2), 503–519 (2020). HBMAEE 10.1002/hbm.24818 31600024 PMC7268021

[50] Watanabe H. et al., “Hemoglobin phase of oxygenation and deoxygenation in early brain development measured using fNIRS,” Proc. Natl. Acad. Sci. 114, E1737–E1744 (2017). 10.1073/pnas.1616866114 28196885 PMC5338505

[51] Punwani S. et al., “Correlation between absolute deoxyhaemoglobin [dHb] measured by near infrared spectroscopy (NIRS) and absolute R2’ as determined by magnetic resonance imaging (MRI),” Adv. Exp. Med. Biol. 413, 129–137 (1997). AEMBAP 0065-2598 10.1007/978-1-4899-0056-2_14 9238493

[r52] Punwani S. et al., “MRI measurements of cerebral deoxyhaemoglobin concentration [dHb]—correlation with near infrared spectroscopy (NIRS),” NMR Biomed. 11, 281–289 (1998). NMRBEF 0952-3480 10.1002/(SICI)1099-1492(199810)11:6<281::AID-NBM529>3.0.CO;2-6 9802470

[53] Strangman G. et al., “A quantitative comparison of simultaneous BOLD fMRI and NIRS recordings during functional brain activation,” NeuroImage 17(2), 719–731 (2002). NEIMEF 1053-8119 10.1006/nimg.2002.1227 12377147

[54] Braver S. L. Thoemmes F. J. Rosenthal R., “Continuously cumulating meta-analysis and replicability,” Perspect. Psychol. Sci. 9(3), 333–342 (2014). 10.1177/1745691614529796 26173268

[r55] Goh J. X. Hall J. A. Rosenthal R., “Mini meta-analysis of your own studies: some arguments on why and a primer on how: mini meta-analysis,” Soc. Personal. Psychol. Compass 10(10), 535–549 (2016). 10.1111/spc3.12267

[56] Valentine J. C. Pigott T. D. Rothstein H. R., “How many studies do you need? A primer on statistical power for meta-analysis,” J. Educ. Behav. Stat. 35(2), 215–247 (2010).1076-9986 10.3102/1076998609346961

