# Supplementary material for: Understanding variability in full-term newborns’ fNIRS data: the impact of birth weight and gestational age on infants’ speech perception abilities
Source: Neurophotonics. 2026 Jul 6;13(3):035001. doi: 10.1117/1.NPh.13.3.035001 (PMC13336347; doi:10.1117/1.NPh.13.3.035001)
Supplement: Supplementary file 1 [file NPh_013_035001_SD001.pdf]

Understanding variability in full-term newborns' NIRS data: the impact of birth weight and gestational age on infants' speech perception abilities

Noémi Szeberényi [a,d], Judit Gervain [a,b,c], Jessica Gemignani [a,b,*]
[a]University of Padua, Department of Developmental Psychology and Socialization, Padua, Italy
[b]Padova Neuroscience Center, Padua, Italy
[c]Université Paris Cité & CNRS, Integrative Neuroscience and Cognition Center, Paris, France
[d]Budapest University of Technology and Economics, Department of Cognitive Neuroscience, Budapest, Hungary

Supplementary Material

1. Methods

1.1 Whole-head single channel analyses

In addition to the analyses presented in the main manuscript, activation values and effect sizes were also extracted, in the same fashion as described in Section 2.2.2, from each channel, as opposed to ROIs as in the analyses reported in the main text.

For each of the three comparisons of interest (Condition 1 vs. 0; Condition 2 vs. 0; Condition 2 vs. Condition 1) model selection was performed as described in Section 2.2.4.1. First, each channel was labelled according to their cortical localization, following anatomical labelling provided in Abboub et al., 2016 (8 channels distributed bilaterally on the temporal areas, 8 on the frontal areas, 8 on the parietal areas). Then random-effects-only models were fit with random intercepts for SubjectID, SubjectID:ROI:Hemisphere, Channel, StudyID, and Lab. Among the ones that converged, we selected the random effects structure yielding the lowest Akaike Information Criterion (AIC). We then selected the fixed effects for factors Age in Days, Gestational Age at birth, Birth Weight, ROI (Temporal / Frontal / Parietal) and Hemisphere (LH/RH), as well as their two-way or three-way interactions. The model with the lowest AIC value were selected as the best-fitting model. When multiple models had comparable AIC values, we selected the most parsimonious model. The results are shown in Table S1.

1.2 ROI-based single channel analyses

We applied the same analysis restricted to individual channels contained in the ROIs (LH frontal ROI: channels 2, 5; RH frontal ROI: channels 13, 15; LH temporal ROI: channels 3,6; RH temporal ROI: channels 17,19). The results are shown in Table S2.

2. Results

Tables S1 and S2 summarise models and effects found for each analysis and hemoglobin component. Figures show all significant post hoc comparisons.

2.1 Whole-head single channel analyses

	HbO	*HbR*
Condition 1	Hemisphere<br><br>• ns	Hemisphere, ROI<br><br>• Main effect of **Hemisphere**: F(1, 936) = 5.68, p < 0.05; LH-RH: mean difference = 0.00177, SE = 0.000744, df = Inf, t = 2.383, p = 0.0172<br>• Main effect of **ROI**: F(2, 953) = 8.84, p < 0.001; Frontal – Temporal: mean difference = 0.00383, SE = 0.000913, df = Inf, t = 4.201, p < 0.0001
Condition 2	GA<br><br>• ns	BW, ROI<br><br>• Main effect of **ROI**: F(2, 936) = 5.83, p < 0.01; Frontal – Temporal: mean difference = 0.00298 , SE = 0.000874, df= Inf, t= 3.409, p= 0.0019
Condition 1 vs 2	GA<br><br>• ns	BW<br><br>• ns<br>•

Table S1: Summary of the best fitting models and corresponding effects for each analysis using single channels from the whole array

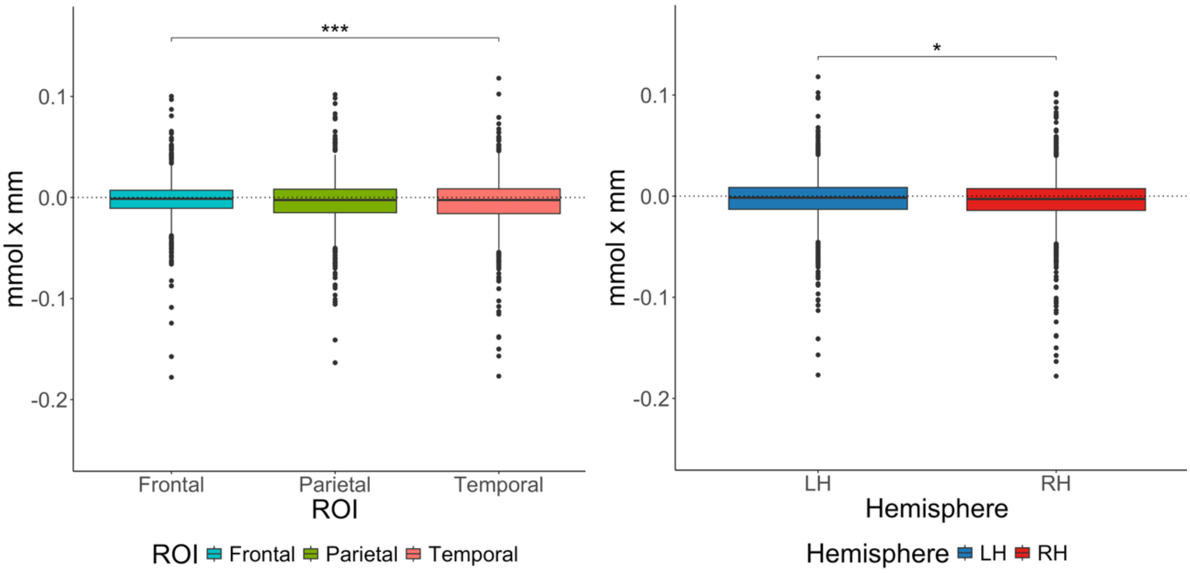

Figure S1: Main effects of ROI (left) and Hemisphere (right) in the whole-head single channel analysis over HbR in Condition 1

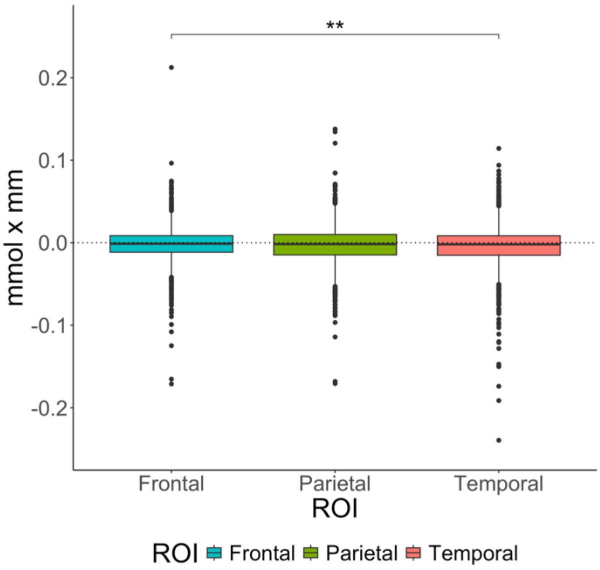

Figure S2: Main effect of ROI in the whole-head single channel analysis over HbR activations in Condition 2

2.2 ROI-based single channel analyses

	HbO	*HbR*
Condition 1	ROI<br><br>• Main effect of **ROI**: F(1, 507) = 13.8, *p* < 0.001; Frontal – Temporal: mean difference = -0.0105, SE = 0.00282, df = 514, t = -3.713, p = 0.0002	ROI<br><br>• Main effect of **ROI**: F(1, 526) = 6.89, p < 0.01; Frontal – Temporal: mean difference = 0.00314, SE = 0.0012, df = 526, t = 2.625, p = 0.0089

Condition 2	ROI<br><br>• Main effect of **ROI**: F(1, 499) = 10.39, *p* < 0.01; Frontal – Temporal: mean difference = -0.00885, SE = 0.00275, df = 523, t = -3.222 , p = 0.0014	ROI<br><br>• Main effect of **ROI**: F(1, 499) = 3.92, *p* < 0.05; Frontal – Temporal: mean difference = 0.00212, SE = 0.0011, df = 517, t = 1.98, p = 0.0483
Condition 2 vs 1	GA<br><br>• ns	BW, GA, ROI, BW:ROI, GA:ROI<br><br>• Main effect of **ROI:** F(1, 481) = 7.47, p < 0.01; Frontal – Temporal: mean difference = 0.00392, SE = 0.0314, df = 508, t = 0.125, p = 0.9009 (ns)<br>• Interaction **BWxROI:** F(1, 523) = 9.1, p < 0.01; Frontal – Temporal, slope difference = -0.000283, SE = 9.42e-05, df = 533, t = -3.003, p = 0.0028<br>• Interaction **GAxROI:** F(1, 487) = 11.1, p < 0.001; Frontal – Temporal, slope difference = 0.0134, SE = 0.00402, df = 495, t = 3.327, p =0.0009

Table S2: Summary of selected models and corresponding effects for each analysis, using single-channels from the Temporal and Frontal regions of interest.

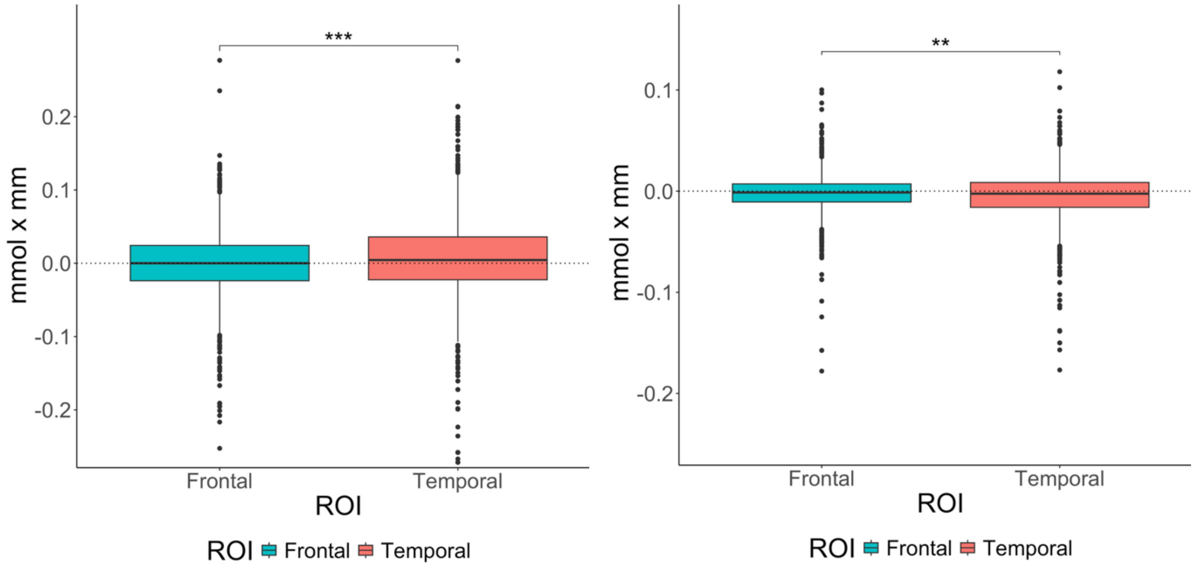

Figure S3: Main effects of ROI in ROI-based single channel analysis over HbO (left) and HbR (right) activations in Condition 1

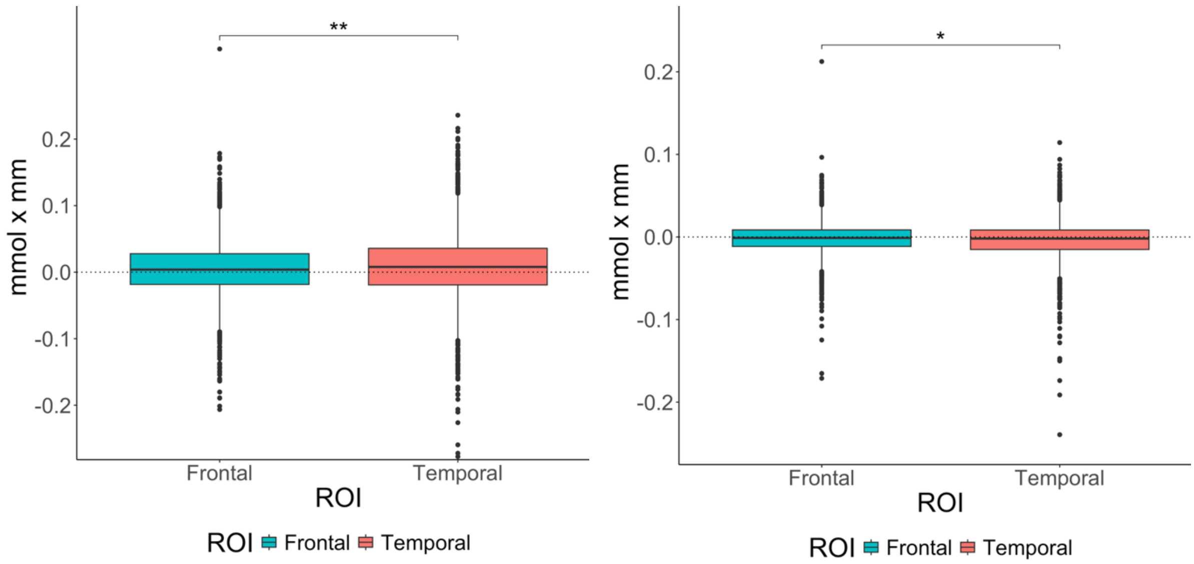

Figure S4: Main effects of ROI in ROI-based single channel analysis over HbO (left) and HbR (right) activations in Condition 2

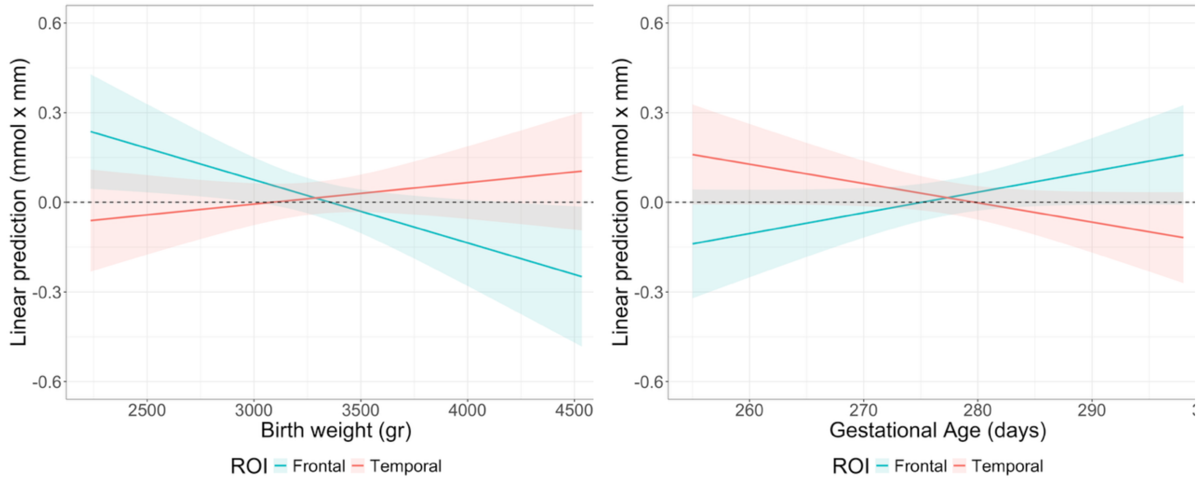

Figure S5: (Left) The interaction of BW and ROI showing a significantly more positive slope in the temporal than in the frontal areas for the effect sizes of the differential response between Conditions 1 and 2 in HbR. (Right) The interaction of GA and ROI showing a significantly more positive slope for the frontal than for the temporal areas for the effect sizes of the differential response between Conditions 1 and 2 in HbR.
